# Consolidation of Hierarchy-Structured Nanopowder Agglomerates and Its Application to Net-Shaping Nanopowder Materials

**DOI:** 10.3390/ma6094046

**Published:** 2013-09-16

**Authors:** Jai-Sung Lee, Joon-Phil Choi, Geon-Yong Lee

**Affiliations:** Department of Metallurgy and Materials Science, Hanyang University-ERICA, Ansan, 426-791, Korea; E-Mails: jpchoi84@hanyang.ac.kr (J.-P.C.); kylee0809@hanyang.ac.kr (G.-Y.L.)

**Keywords:** hierarchical structure, consolidation, nanopowder agglomerates, net-shaping

## Abstract

This paper provides an overview on our recent investigations on the consolidation of hierarchy-structured nanopowder agglomerates and related applications to net-shaping nanopowder materials. Understanding the nanopowder agglomerate sintering (NAS) process is essential to processing of net-shaped nanopowder materials and components with small and complex shape. The key concept of the NAS process is to enhance material transport through controlling the powder interface volume of nanopowder agglomerates. Based upon this concept, we have suggested a new idea of full density processing for fabricating micro-powder injection molded part using metal nanopowder agglomerates produced by hydrogen reduction of metal oxide powders. Studies on the full density sintering of die compacted- and powder injection molded iron base nano-agglomerate powders are introduced and discussed in terms of densification process and microstructure.

## 1. Introduction

The potential application of nanoscale materials as novel structural or functional engineering materials largely depends on the consolidation of powders into bulk nanoscale solids. In general, pressure-assisted sintering is known to be adequate for both reaching full density and preventing grain growth [1]. Besides the fabrication of nanomaterials into engineering components by hot pressing or sinter-forging, another promising objective is the application of nanopowders to the net-shaping technique as powder injection molding (PIM). In this case, the consolidation of the net-shaped powders is only achieved by pressureless sintering.

It was reported that Fe base nanopowder compacts underwent the densification process strongly depending on the state of powder agglomeration [2]. The consolidation was incomplete due to a severe influence of particle agglomeration. Consequently, full densification of nanopowder compact even at elevated temperature could be achieved by optimizing the agglomerate size through either high-pressure compaction or reducing agglomerate size. Based upon these findings, Lee *et al.* [3,4,5] suggested a new sintering concept for consolidation of nanoscale powders, so called nanopowder agglomerate sintering (NAS) process.

The key concept of the NAS process is based on the optimization of structure design and full density processing of nanopowder into nanostructured micro-components. The kinetics of this process is found to be controlled by material transport through hierarchical interface structures of nanopowder agglomerates [5,6,7]. From the studies of densification process [2,5,6,7,8] and diffusion process [9,10,11] in Fe–Ni nanomaterials, it has been reported that the hierarchy-structured grain boundaries consisting of nano grain boundary and agglomerate boundary act as high diffusion paths for densification process. In particular, it was found that atoms diffuse much faster along agglomerate boundaries compared to nano grain boundary.

This experimental finding provides a breakthrough for processing full density nanopowders by optimal design of the agglomerate structure, *i.e.*, hierarchical interfaces in nanopowders. Moreover, based on this processing concept, Lee and his coworkers [3,4,5] fabricated nanograin structured Fe base PIM parts with full density and superior mechanical property owing to grain refinement effect. This is a good example or the application of NAS process to processing a net-shaped powder material. Another application example of NAS process is recently reported for the fabrication of full density Fe nanomaterial [12].

In this paper we review our recent investigations on the consolidation of hierarchy-structured nanopowder agglomerates in terms of the densification process and microstructural development and also introduce on their application studies for processing a net-shaped Fe base nanopowder parts using PIM technology and pressureless sintering. In addition, current research on low-temperature PIM using hierarchy-structured micro-nano powder feedstock is briefly addressed.

## 2. Optimal Processing of Iron Base Nanopowder Agglomerates

**Figure 1:**
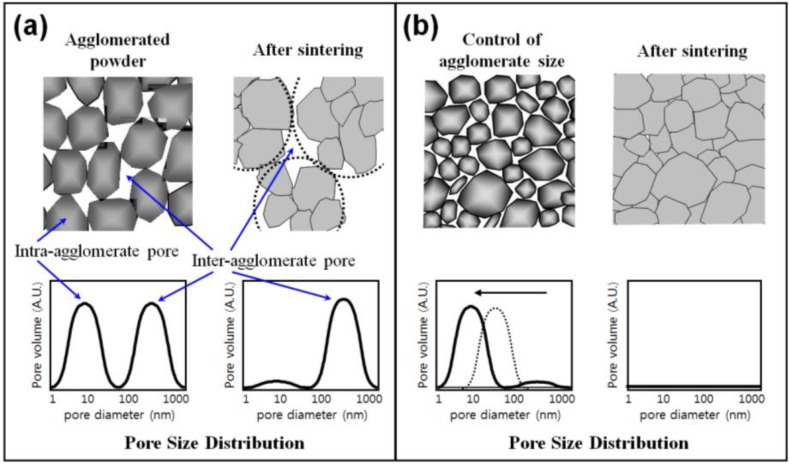
Schematic illustration of pore size distribution in agglomerated nanocrystalline powder (**a**) without and (**b**) with controlling of distribution of agglomerate size [13].

Figure 1 shows a schematic illustration of densification process by controlling of agglomerate size [13]. The agglomerate powder compact exhibits a bimodal pore distribution comprised of nanoscale intra-agglomerate pores and micron scale inter-agglomerate pores. While the nano-sized intra-agglomerate pores are easily eliminated in the course of sintering, the micro-sized inter-agglomerate pores remain and restrain densification (Figure 1a). After controlling of agglomerate size distribution, the compact part has homogeneous- or narrow pore size distribution (Figure 1b) and can reach full densification after sintering. The structural modification of the agglomerated nanopowders by controlling agglomerate size might be a key technology to obtain smaller and narrower pore size distribution. This chapter describes the full density processing of net-shaped Fe base nanopowder agglomerates by controlling agglomerate size in terms of densification process and microstructure.

**Figure 2:**
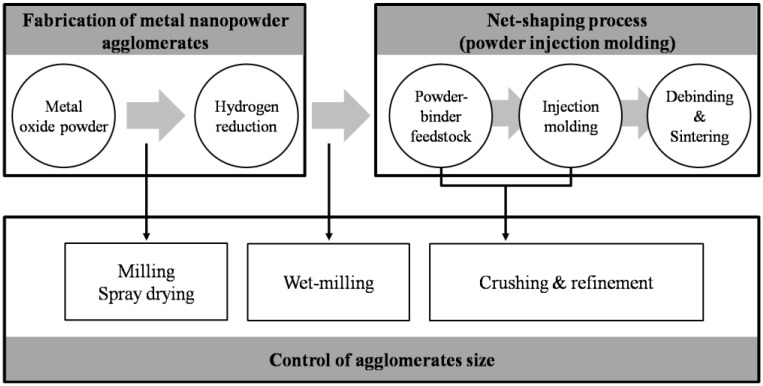
Experimental procedure for synthesis and net-shaping process of metal nanopowders produced by hydrogen reduction of ball-milled oxide powder [3].

Figure 2 schematically represents the experimental procedure utilized for net-shaping process of Fe base nanopowder [3]. The process involves a two-step procedure consisting of Fe base nanopowder synthesis by hydrogen reduction of oxide precursors [14,15,16,17] and full densification process of PIM nanopowder by pressureless sintering. As depicted in the diagram, the large inter-agglomerate pores should be efficiently minimized by using different processing routes which can control the agglomerate size during the entire process. Intuitively, two methods for controlling agglomerate size and distribution are suggested; one is the wet milling of as-reduced nanopowder in dispersant containing solution [3,5,18] and the other is the mixing of nanopowder with the binder material for making PIM feedstock [3,5,18,19]. The wet-milling of nano-agglomerate powder in non-oxidation atmosphere can lead to increasing of packing density of agglomerate powder owing to improvement of particle size uniformity [20]. The maximum packing density for random packing of mono-sized spheres is predicted to be independent of the sphere size, and this prediction has been verified experimentally [21].

**Figure 3:**
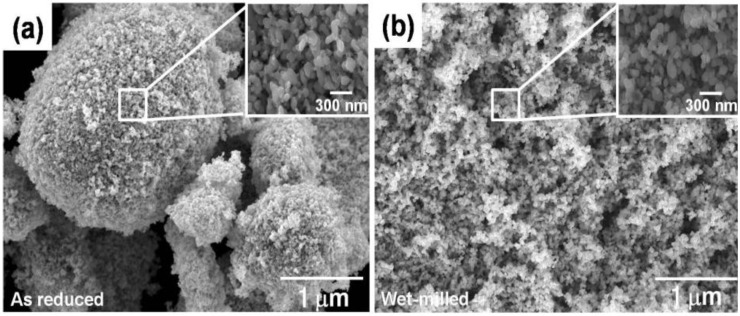
SEM micrographs of (**a**) as-reduced and (**b**) wet-milled Fe nanopowder [18].

Figure 3a,b show the SEM (scanning electron microscope) micrographs of Fe nanopowders after wet-milling in a solution of stearic acid dissolved alcohol in comparison with that for as-reduced state. After wet-milling, the Fe nano-agglomerate powders consisting of Fe particles with 100 nm in size had an agglomerate size distribution ranging from 0.5 to 5 μm, reflecting a more homogeneous distribution than that for the as-reduced powders. It was also observed that the Fe particles with 100 nm in size did not change during wet-milling process [12,18]. The same result of this wet-milling effect on agglomerate size was also obtained in other Fe base nanopowders [5].

**Figure 4:**
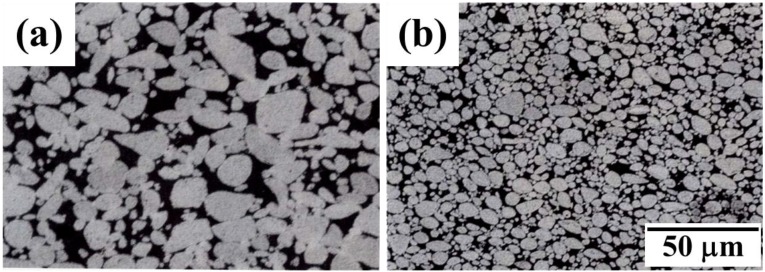
Microstructure of (**a**) the die-pressed compact and (**b**) the powder injection molding (PIM) brown part of Fe–40 wt % Ni nanopowder [19].

Regarding the other method for making PIM feedstock, the mixing process of Fe–Ni nanopowder and binder material for preparing the PIM feedstock turned out to be effective for uniform and isotropic sintering process, resulting in homogeneous microstructure [19]. As depicted in Figure 4, the PIM part shows much finer and more homogeneous microstructure (Figure 4b) compared to the conventional powder compact (Figure 4a). Such discrepancy in the powder structure is attributed to the agglomerate disintegration occurred during preparation of feedstock by mixing of Fe–Ni nanopowder and binders. This argument was supported by the result of pore size distribution showing smaller volume of inter-agglomerate pore and larger volume of intra-agglomerate pore compared to the conventional compact.

## 3. Effect of Hierarchical Gb Structure of Nanopowder Agglomerates on Densification Kinetics and Microstructure

### 3.1. Diffusion-Controlled Densification Process

**Figure 5:**
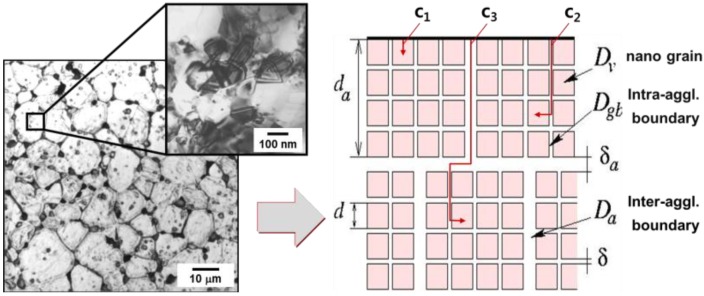
Microstructure of the Fe–40 wt % Ni nanomaterial observed by optical and transmission electron microscope and a model of nanomaterials composed of hierarchical microstructure consisting of nano- and agglomerate boundaries [10].

The key issue for the application of nanopowder to engineering parts is how to consolidate the powder into the sintered part with full density and nano-grained microstructures, especially by pressureless sintering [22]. As described above, it was reported that the bimodal type of grain boundaries in Fe–Ni nanopowder alloy consisting of nano-sized grain boundary and agglomerate boundary act as high diffusion paths for densification process (Figure 5). The activation energies of nano grain- and agglomerate boundary diffusion are listed in Table 1.

**Table 1:** Arrhenius parameters of Fe and Ni diffusion along nano-GBs and inter-agglomerate boundaries in nanocrystalline Fe–Ni alloy [11].

Element	nano-GBs		aggl. boundaries	
	D_0_ (m^2^/s)	Q_gb_ (kJ/mol)	D_0_ (m^2^/s)	Q_a_(kJ/mol)
Fe	4.2 × 10^−3^	187	3.4 × 10^−3^	148
Ni	9.4 × 10^−4^	177	1.9 × 10^−3^	134

Nano grain boundary diffusivity was calculated to be similar to that for coarse-grained materials [23]. This fact is explained by the circumstance that during the production of Fe–40 wt % Ni nanoalloy by the powder metallurgical method and subsequent sintering, grain growth occurred and the grain boundary have relaxed to equilibrium grain boundary structures. Comparing the diffusion data (Table 1) with that of the densification process, activation energies for intra-agglomerate boundary- (187 kJ/mol for Fe and 177 kJ/mol for Ni) and inter-agglomerate diffusion (148 kJ/mol for Fe and 134 kJ/mol for Ni) approximately correspond to that for the intermediate sintering stage of 85~90% (120~200 kJ/mol) [8]. This implies that densification process of nanocrystalline Fe–40 wt % Ni powder is initiated by the diffusion process along high diffusion paths of intra-agglomerate- and inter-agglomerate boundaries.

In order to examine the role of agglomerate boundary of hierarchical structured nanopowder in sintering kinetics, densification kinetics for Fe–8 wt % Ni nanopowder agglomerate compacts with different agglomerate size of 5 and 500 μm was investigated in terms of the activation energy for densification process [24]. The parameters required to calculate activation energy on the basis of Equation (1) were obtained from the result of densification process during heat-up sintering at various heating rates:

(1) $$\ln(\frac{\phi }{T^{2}})=\ln(\frac{CR}{Y^{n}Q})-\frac{Q}{RT}$$

where *ϕ* is the ratio of the heating rate, *T* is temperature, *R* is gas constant, *Y* is the identical value of shrinkage and *Q* is activation energy for densification [24].

Both agglomerate powders were virtually identical, only differing in the agglomerate size. To eliminate inter-agglomerate pores, both powders were equally compacted to 72% of theoretical density (T.D.) with compacting pressure of 1.2 GPa.

**Figure 6:**
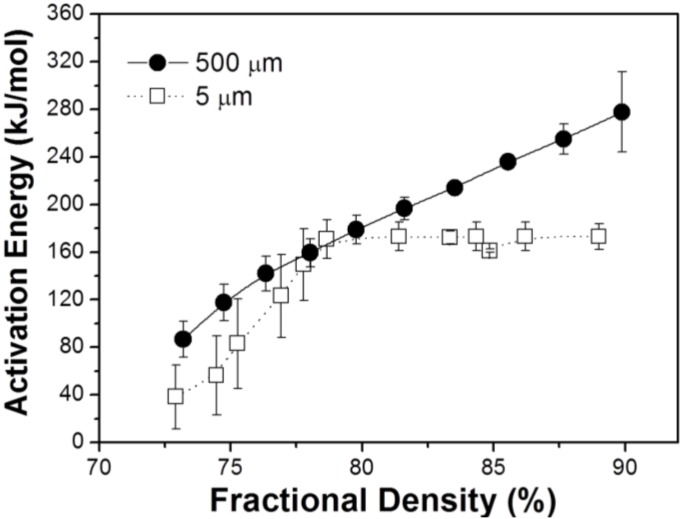
Apparent activation energy for densification process for Fe–8 wt % Ni nanopowder agglomerates during sintering at intermediate temperatures [24].

Figure 6 compares the dependence of apparent activation energies for densification process on fractional sintered density. Apparent activation energy for the nanopowder compact with 500 μm size sample increased from 80 to 280 kJ/mol as densification proceeded from 72% to 90% T.D. On the other hand, in the case of 5 μm sample, activation energy increased gradually from 40 to 170 kJ/mol with sintering from 72% to about 80% T.D. and thereafter retained 170 kJ/mol even above 80% T.D. Considering the agglomerate size in both samples, it is expected that the large size agglomerate sample requires higher activation energy for sintering of nanoparticles in the agglomerate compared to the small agglomerate sample. This is because the sintering process takes place mainly and dominantly inside the agglomerates. Namely, densification and grain growth can occur simultaneously until the grain size reaches the agglomerate size. Therefore the large agglomerate sample showing a great change of activation energy (80 to 280 kJ/mol) is thought to undergo densification process by various diffusion processes from surface (60 kJ/mol), interface (180 kJ/mol) to volume diffusion (280 kJ/mol) [10,11]. This argument might be reasonable when the activation energy for densification process is compared with that for diffusion process. This implies that the sintering kinetics controlled by densification and grain growth inside the agglomerate can be explained in terms of all kinds of diffusion mechanisms. On the contrary, it seems that the sintering of the small agglomerate sample is enhanced owing to larger fraction of inter-agglomerate boundary. Consequently, the optimal sintering route for nanopowder is considered by reducing the agglomerate size, *i.e.*, enlarging the agglomerate boundary volume, which provides faster diffusion path [11].

### 3.2. Densification and Microstructure

**Figure 7:**
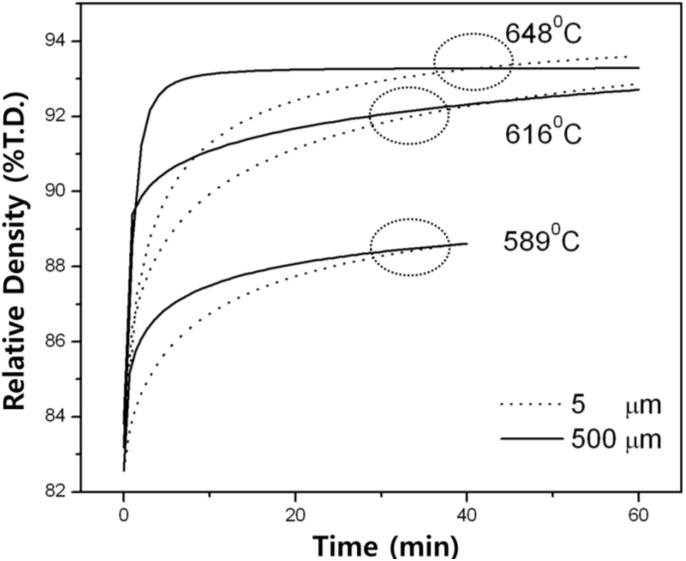
Densification process for Fe–40 wt % Ni nanopowder agglomerates during sintering at intermediate temperatures [6].

Figure 7 compares the densification processes of Fe–40 wt % Ni nano-agglomerate powder compacts of two different agglomerate sizes during isothermal sintering at intermediate temperatures of 589, 616 and 648 °C [6,7]. It is seen that the large agglomerate sample of 500 μm agglomerate underwent rapid densification process in the initial stage of sintering while the gradual and lower densification process proceeded in the 5 μm agglomerate. However, it is noted that at a certain sintering time the densification, the process became inverse. As a result, after this, the small agglomerate powder compact could have higher density. It seems that such an interesting densification behavior occurs more or less earlier at higher temperatures.

The inverse behavior in the densification processes for two different agglomerate sizes can be explained in terms of powder packing structure and pore structure. Regardless of the same porosity in two compacts, the small agglomerate sample forms a higher volume fraction of inter-agglomerate boundary rather than that of intra-agglomerate boundary. Therefore, the 5 μm agglomerate can have much higher volume of atom diffusion path for rapid material transport for densification than the large agglomerate sample. However, in terms of driving force for sintering, which can be expressed as sintering reaction site, rather such large volume of the agglomerate boundary plays a negative role in sintering of nanopowder which limitedly proceeds within the agglomerates. Initially dominant sintering takes place first in the agglomerate, then followed by sintering between neighbored large powders as locally full densified agglomerate powder. At this point when the neighboring sintered agglomerate powders start to sinter together or neighboring nanopowders near the boundaries sinter we can expect further continuous densification process in the sample leading to eventually inverse densification in later stage of sintering.

**Figure 8:**
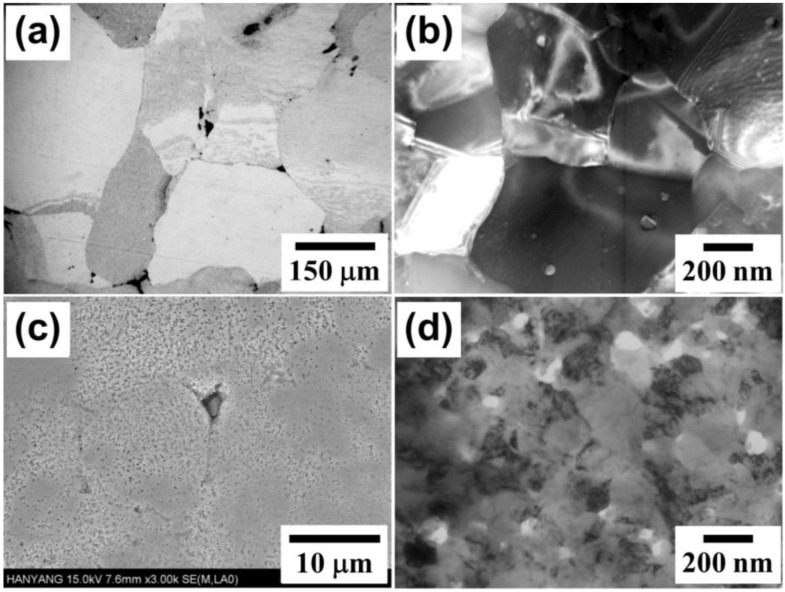
Microstructures of hierarchical structured Fe–40 wt % Ni nanopowder agglomerates sintered at 616 °C for 2 h: (**a**,**b**) large agglomerate powder and (**c**,**d**) small agglomerate powder.

An evidence of agglomerate boundary acting as effective diffusion path for densification process is confirmed by microstructure observation. First, in case of the large agglomerate sample in Figure 8a, the agglomerate boundaries did not migrate during sintering with keeping the boundaries in as the compacted state. It is also observed that large inter-agglomerate pores exist mostly at triple junctions of agglomerates. About a 10% density increment of the whole large agglomerate powder resulted from the sintering of Fe–Ni nanopowders inside the agglomerate. But they are hardly densified under the present sintering condition due to low driving force as long as pores at triple junctions of agglomerates are eliminated. Therefore further full densification of the large agglomerate powder compact is unlikely achieved under this low temperature sintering condition. Contrary to the agglomerate boundary, nano boundaries seem to be relaxed in equilibrium state after grain growth. This is supported by TEM microstructure observation of Figure 8b. The nano grains of ~100 nm in initial size grew about 4 times to 400 nm after sintering at 616 °C for 2 h.

Apart from the large agglomerate sample, the small agglomerate powder compact shows a different microstructural development as seen in Figure 8c. Interestingly some distinguishable agglomerates of which structures seemingly correspond to the initial agglomerate powders in size and shape could be detected. However, also most of the boundaries look like destroyed and collapsed boundaries. As the sintering of nanopowders inside the agglomerates proceeds in the small agglomerates the densification process reaches the barrier boundaries earlier, followed by retarding densification. However, large numbers of nanopowders in the agglomerate boundary region try to sinter together for further densification process. This might be responsible for partly disappearance of the boundaries.

Accordingly, sintering of the 5 μm agglomerate Fe–Ni nanopowder compact is thought to proceed by two competing sintering processes; one is the sintering of nanopowder within the agglomerates and the other is that of agglomerates. The latter process can only take place when the particles neighboring in boundaries have sufficient driving force for further continuous sintering process. For this reason the small agglomerate sample might undergo slow densification in early stage of sintering due to the hindering effect of the agglomerate boundary and then gradual higher densification at such critical point as described. TEM micrograph of the 5 μm agglomerate sample (Figure 8d) clearly confirms this expectation. The microstructure consists of smaller nanograins less than 200 nm and nano pores which are located at grain boundaries and triple junctions. This microstructural feature supports the inverse densification in the 5 μm agglomerate sample of Figure 7. This is an evidence of slow sintering effect of the 5 μm agglomerate. During the structural homogenization of agglomerates such microstructure of Figure 8d provides potential densification driving force which leads to an inverse effect.

## 4. Application of NAS to Fabrication of Iron Base Powder Material Components

In order to check the applicability of the NAS process to net-shaping of nanopowders small double gear component as well as die compact sample was fabricated by die compaction and powder injection molding of Fe base nanopowder and Fe micro-nano powder feedstock of which agglomerate size was optimized by wet-milling and powder injection molding.

### 4.1. Full Density Processing of Fe Nanopowder by NAS Process

**Figure 9:**
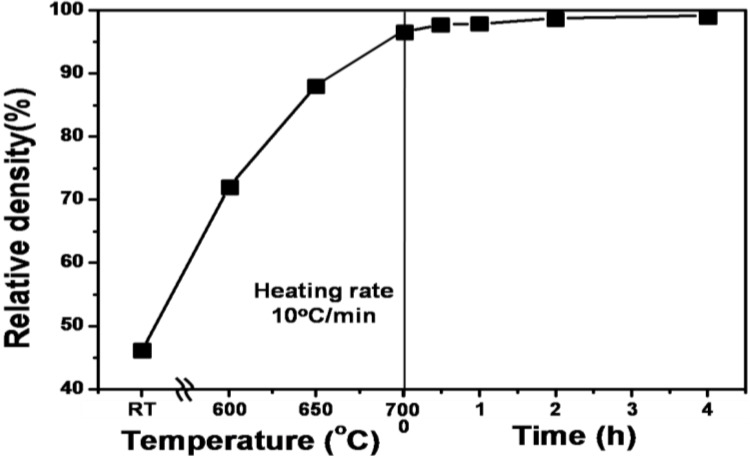
The densification behavior of Fe nanopowders with optimal agglomerate size during heat-up with a heating rate of 10 °C/min and subsequent isothermal sintering at 700 °C in a hydrogen atmosphere [12].

Figure 9 shows the densification process of the wet-milled Fe nanopowders during heat-up with a heating rate of 10 °C/min and subsequent isothermal sintering at 700 °C in a hydrogen atmosphere [12,18]. It is seen that remarkable densification occurs in low temperature sintering, especially during heat-up stage reaching near full density of 96.5% T.D. at 700 °C. Considering the amount of densification occurring during heat-up sintering only, it is thought that the heat-up sintering process includes entire stages of sintering Fe nanopowders from early stage to final stage, mostly intermediate stage of sintering. This result is quite different from the case for large agglomerate nanopowders which are larger than a few 10 μm and not wet-milling treated powders. As described above, this is basically due to a difficult-to-sinter bimodal pore distribution, which is due to the coexistence of intra- and inter-agglomerate pores. Based upon the previous works [2,5,6,7,8], the occurrence of full densification during heat-up process in Figure 9 is related to NAS process in which the sintering kinetics are governed by material transport through the hierarchical interface structures of the nanopowder agglomerates. This phenomenon was explained by SEM micrographs for microstructural homogenization occurring during heat-up sintering.

**Figure 10:**
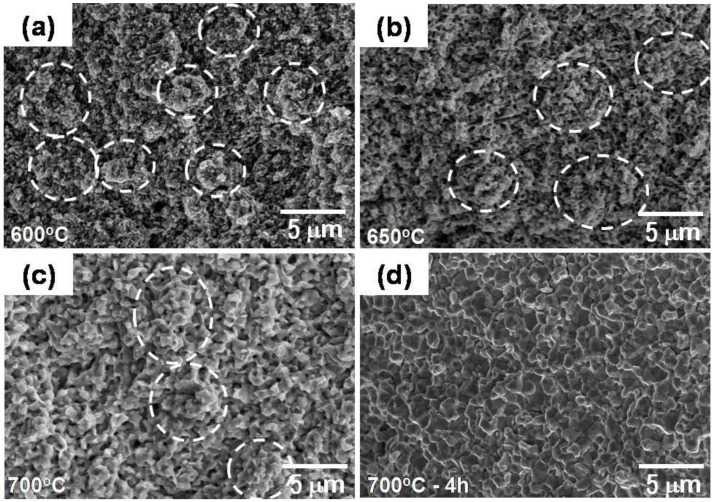
SEM fractographs of Fe nanopowders showing microstructural homogenization during heat-up sintering with a heating rate of 10 °C/min up to (**a**) 600 °C, (**b**) 650 °C, (**c**) 700 °C and (**d**) sintered at 700 °C for 4 h, for comparison. * White dotted circles denote agglomerates [12].

As seen in Figure 10a–c, the agglomerates with its initial size ranging from 0.5 to 5 μm enlarged with increasing densification process and as a result, the agglomerate boundaries gradually disappeared. Initially dominant sintering takes place first in the agglomerate, followed by sintering between neighbored large powders as locally densified agglomerate powders. Finally, fully-densified homogeneous microstructure, as shown in Figure 10d, is obtained. In the aspect of sintering kinetics based on diffusion process, the powder interfaces consisting of hierarchical interfaces of agglomerate- and nanograin boundaries act as diffusion paths for material transport during such rapid densification process. This argument has been also confirmed by our previous studies using Fe–Ni nanopowder agglomerates on densification process during heat-up sintering as well as isothermal sintering [2,3,5,6,7,8]. It is interesting that the pure Fe nanopowder compact with optimal agglomerate size undergoes remarkable densification during heat-up sintering as well as isothermal sintering at low temperature of 0.53T_m_ (melting point) of Fe. In particular, it should be noted that the densification in isothermal sintering reaches near full density in the range of 96.5% to 99.2%. This indicates that the isothermal sintering process in this study corresponds to final stage of sintering [25].

**Figure 11:**
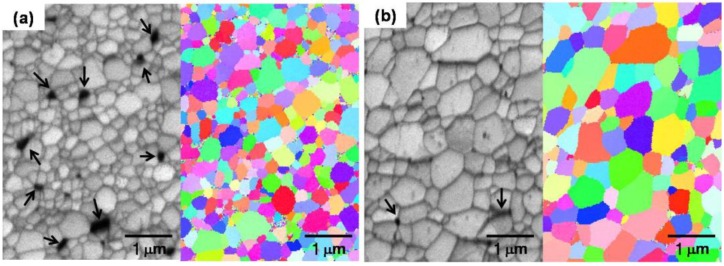
SEM micrographs and EBSD (electron backscatter diffraction) images of pure Fe nanopowders sintered at 700 °C for **(a)** 0 h and **(b)** 4 h. * The arrows indicate pores mostly locating at grain boundaries and triple junctions [12].

Final stage of sintering process is shown in the results of SEM-EBSD (electronback scatter diffraction) of Figure 11. The SEM micrographs clearly show that fine pores exist mostly at grain boundaries and triple junctions in the final sintering stage. The EBSD results also represent that the microstructure of sintered Fe nanopowder consists of different colored Fe grains indicating different crystallographic orientations. This is direct evidence that the sintered Fe sample in this study is composed of high angle grain boundary structure. It is very interesting that fine pores still exist at grain boundaries and triple junctions even in the final stage of sintering process in this study. Due to the pore at interfaces, enhanced densification process can last until it reaches final full density even in low temperature sintering. This is because the grain boundary acting as a high-diffusion path for material transport in the intermediate sintering at low temperature could be efficiently incorporated with the pores in the entire sintering process.

**Figure 12:**
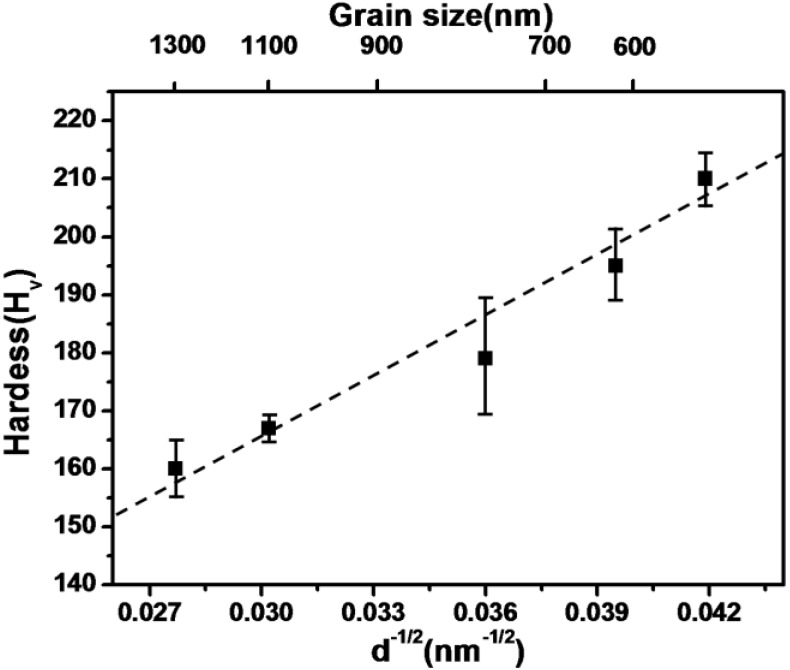
The variation of Vickers hardness (H_v_) of Fe nanopowder parts sintered at 700 °C as a function of d^−1/2^ [12].

It is interesting that no drastic grain growth takes place during isothermal sintering at 700 °C. As depicted in Table 2, the average grain size was increased by about 1.7 times from 570 nm to 1.3 μm during 4 h of sintering. It is intuitive that pore pinning effect is mostly responsible for such a slow grain growth. To examine mechanical property, Vickers hardness (H_v_) was measured after various sintering times and summarized in Table 2. It is seen that the hardness value decreased with increasing sintering time from H_v_ of 210 at 0 h to H_v_ of 160 at 4 h. However, these values are higher than the values for conventional PM (powder metallurgy) steel parts containing carbon content. This remarkable strengthening effect of the pure Fe nanopowder without the presence of carbon is well interpreted in terms of grain refinement effect. Figure 12 shows the dependence of hardness value on grain size which represents Hall-Petch relationship. It is clearly seen that the hardness values are in a good agreement with a normal Hall-Petch relationship. This means that the strengthening effect by grain refinement described above holds in this grain size range. These results are indicative of promising applications for high strength pure Fe powder materials.

**Table 2:** The sintering properties of Fe nanopowder agglomerate compacts isothermally sintered at 700 °C [11].

Composition	Heating time (h)	Relative density (%)	Hardness (H_v_)	Grain size (μm)
Fe	0	96.3	210	0.57
	0.5	97.6	191	0.64
	1	97.7	176	0.77
	2	98.6	167	1.1
	4	99	160	1.3

### 4.2. Powder Injection Molding of Fe Base Nanopowder Agglomerates

**Figure 13:**
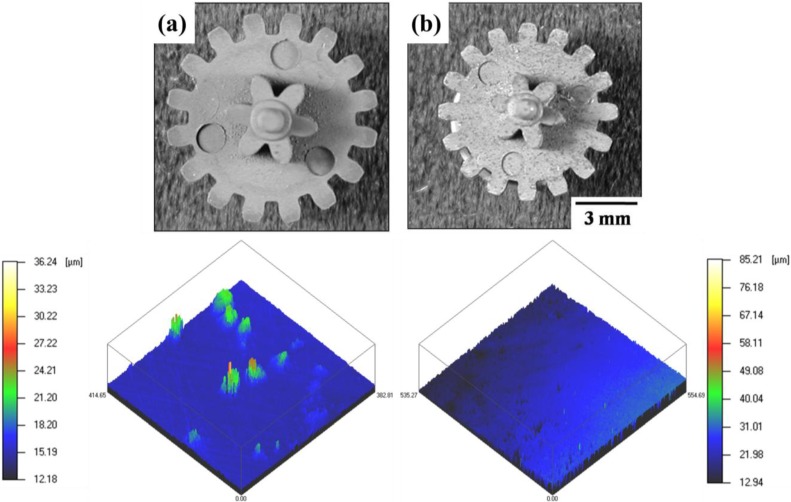
Photographs of the PIM Fe–8 wt % Ni double-gear parts and their 3-dimensional images for surface roughness by AFM: (**a**) brown part and (**b**) sintered part (sintered at 1000 °C for 1 h) [5].

Figure 13 shows the shape and surface roughness of the PIM gear of Fe–8 wt % Ni wet-milled powder after debinding and sintering (sintered at 1000 °C for 1 h) [5,21,26]. As clearly seen, the PIM part keeps its fine gear-shape with uniform full density and no distortion. This means that isotropic shrinkage occurred during sintering process. Especially, the AFM (atomic force microscope) study revealed that the sintered part represents a very sound surface roughness of 160 nm much finer than the brown part of 0.8 μm, satisfying the requirement for surface roughness for micro-PIM part.

**Figure 14:**
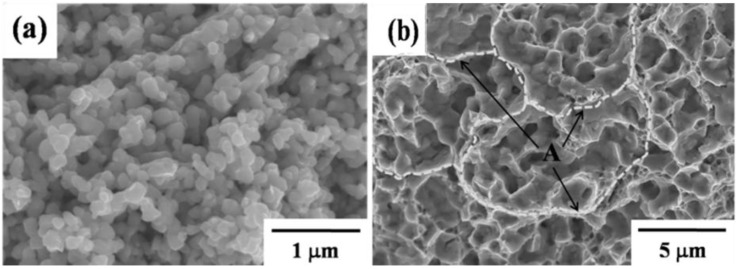
The fracture surface morphologies of (**a**) brown and (**b**) sintered part of PIM Fe–8 wt % Ni nanopowders (A denotes agglomerate boundary) [5].

Figure 14 shows that the wet-milled Fe–8 wt % Ni nanopowder results in a homogeneous part after debinding and sintering. Following debinding, the brown part exhibits a homogeneous and uniform microstructure even though it has a high level of porosity (corresponding to 52% of the pore-free density), as seen in Figure 14a. This uniform and homogeneous microstructure in the brown part is attributed primarily to agglomerate-size control by the wet-milling process. After sintering, the part exhibits a fully densified and nano-grain-coarsened microstructure (~1 μm grain size) (Figure 14b). It is interesting to note that traces of inter-agglomerate boundary are observed on the fracture surface of the sintered part, denoted by white dotted line (Figure 14b). The sintered part of the agglomerated nanopowder consists of micron-sized agglomerate boundaries and nano-sized grain boundaries. This is important evidence of the hierarchical structure of the PIM Fe–8 wt % Ni nanopowder. Densification in Fe–Ni nanopowder proceeds by a diffusion process, preferentially along the two high-diffusivity paths, namely, agglomerate boundaries and nano-grain boundaries [8,10].

**Figure 15:**
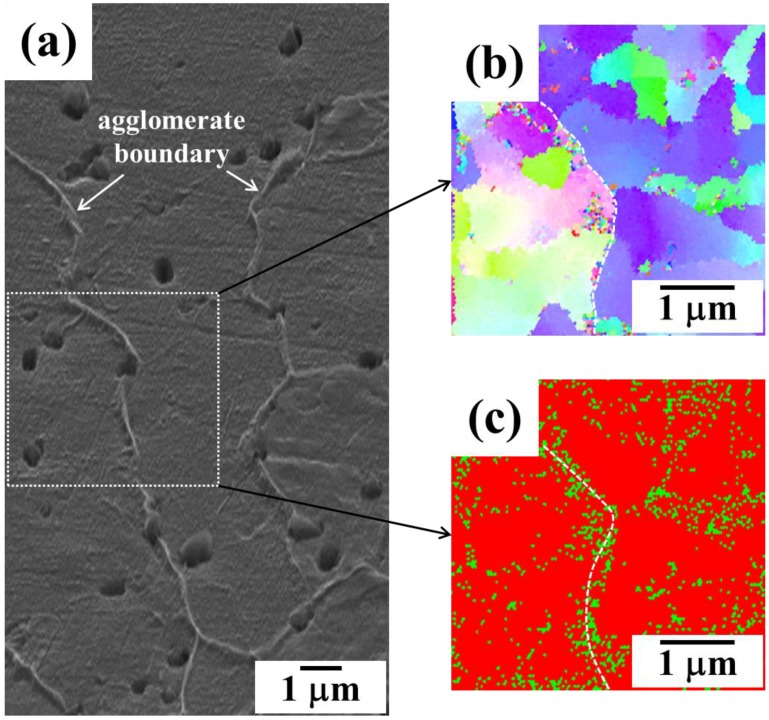
Micrographs of hierarchical structured Fe–8 wt % Ni PIM part showing (**a**,**b**) agglomerate boundary and nano-grains and (**c**) γ-precipitates favorably located at agglomerate boundaries (sintered at 800 °C for 2 h) [5].

Such hierarchical microstructures can be illustrated more clearly by EBSD-images [5]. Figure 15 confirms a hierarchical structure consisting of large agglomerate boundaries and nano-grain boundaries in the same PIM specimen sintered at 800 °C for 2 h. It is also seen that the hierarchical structure of the NAS-processed material results in a phase transformation, namely, the precipitation of γ phase in the α matrix during sintering and subsequent cooling. It is clear that a large number of γ phase precipitates are located on agglomerate boundaries. This phenomenon is evidence that agglomerate boundaries are thermodynamically less stable and structurally unrelaxed than the nano-grain boundaries [3,7,8,9,10,11].

### 4.3. Powder Injection Molding of Bimodal Type Micro-Nano Powder

Recently the authors found that the nano-agglomerate powder can be effectively used for optimal feedstock for micro-PIM in combination with micron powders [27,28]. These works provide new breakthrough for designing optimal feedstock for PIM at low-temperature and low-pressure [29,30]. In this respect it is very important to understand the effect of low temperature enhanced sintering of nanopowder on debinding and subsequent sintering process based on NAS process.

**Figure 16:**
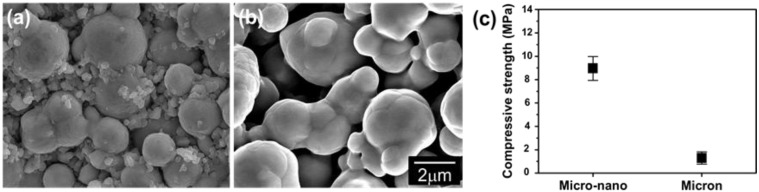
SEM fractographs of the debound parts fabricated using (**a**) Fe micro-25 vol % nano powder; (**b**) Fe micro powder and (**c**) compressive fracture strength of (**a**) and (**b**) [30].

Figure 16 compares the SEM-micrographs of the debound parts of Fe micro-25 vol % nano powder and Fe micro powder sample. While the debound part of the Fe micro powder sample shows locally sintered micro powders and a large interconnected pore, the micro-nano powder sample consists of a micro powder skeleton structure strongly interconnected by nanopowder bridges that are considerably sintered. Generally, the main problem in the spherical shape of micro powder is the formation of a very fragile compact after debinding [31]. This characteristic is seen in Figure 16b. To the contrary, owing to the enhanced sintering effect the micro powders in the micro-nano powder feedstock form a strong skeleton structure effectively by enhanced sintering of nanopowder during the debinding process.

The formation of strong nanopowder bridges during debinding indicates that using high density organic binders with high viscosity is no longer necessary. Instead, some binder materials with lower viscosity and melting temperature can be utilized without any problems in the debinding process. This argument is strongly supported by the result of the green strength measurements by using a compression test [32]. Figure 16c shows that the strength for the micro-nano powder sample (8.96 ± 1.0 MPa) is much higher than that of the micro powder sample (1.29 ± 0.5 MPa) [30]. When considering that there is no difference of the green density in both samples, remarkable strengthening of the green compact after debinding can be explained by strong formation of micro powder skeleton by nano powder bridges.

**Figure 17:**
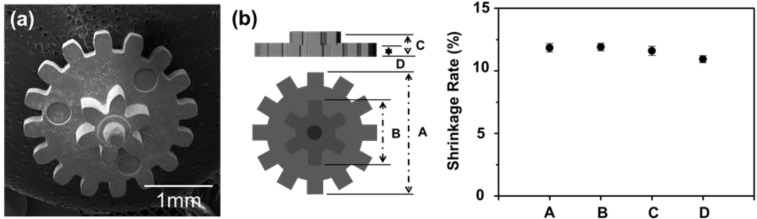
(**a**) SEM micrograph of the sintered double gear fabricated using Fe micro-25 vol % nano powder feedstock and (**b**) linear shrinkage distribution in the sintered part [30].

Figure 17a shows a photograph of the micro-nano powder PIM component after sintering at 1250 °C for 3 h in hydrogen atmosphere. The PIM part keeps a uniform and sound surface structure without deformation or defects. The shrinkage uniformity evaluated by dimensional changes in the percentage in the positions of A, B, C, D of double gear sample before and after sintering is represented in Figure 17b. It is obvious that the micro-nano PIM part underwent isotropic shrinkage during sintering showing an average linear shrinkage of 11.5% ± 0.5%. This shrinkage value fulfills the requirement for micro-PIM part [33].

**Figure 18:**
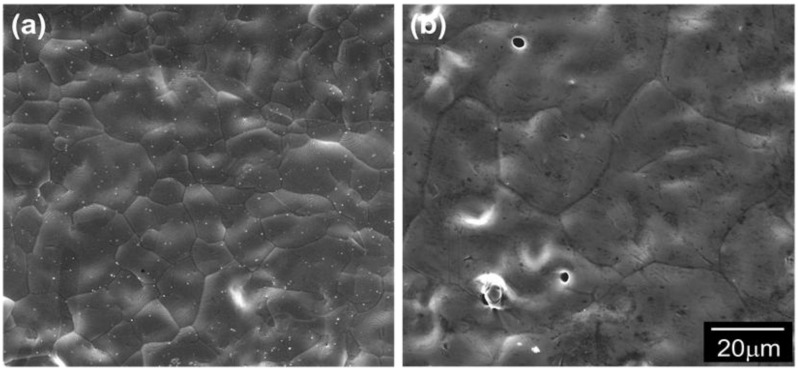
SEM micrographs of the surface of Fe-PIM part sintered at 1250 °C for 3 h, (**a**) micro-nano powder and (**b**) micro powder [30].

Figure 18 shows the SEM-micrograph of the micro-nano PIM part in comparison with that of the micro powder part after sintering. Apparently, the micro-nano powder specimen discloses a fully dense microstructure consisting grains of less than 10 μm in size whereas the micro powder is composed of remarkably coarsened grains at around 80 μm. Such a discrepancy in the microstructure has already been explained in terms of the effect of nanopowder sintering [28]. During the sintering of the micro-nano powders, the micro powders remain almost not-grown, while the nanopowders rapidly grow until their grain size distribution corresponds to that of the micro powders. In addition to this behavior, such the inhibition effect enhances the densification process in sintering of the micro powders. This is due to the fact that the dragging effect of the nanopowder leads to keeping of micro grains which provide material transport paths for rapid densification of the micro powders [28,30,34]. This microstructural feature of the micro-nano PIM part discloses a potential application of the nanopowders to design new sintering technique for processing PIM parts with full density and fine microstructure.

## 5. Conclusions

In this paper we reviewed our recent investigations on the consolidation of hierarchy-structured Fe base nanopowder agglomerates in terms of the densification process and microstructure and also introduced on their application studies for processing a net-shaped parts. Understanding NAS process is essential to processing of net-shaped nanopowder materials and components with small and complex shape. The key concept of NAS process is to enhance material transport through controlling the powder interface volume of nanopowder agglomerates. We found that the enhanced sintering effect at low temperature is an evidence of NAS process which is basically driven by the densification process along hierarchical boundaries consisting of two high diffusivity paths of agglomerate- and nano-grain boundaries. This experimental evidence provides a new idea of full density processing for fabricating micro-PIM part using metal nanopowder agglomerates. It is concluded that the optimization of agglomerate size control of nanopowder is a breakthrough for processing of net-shaped Fe base nanopowder materials.
